# Application of dose kernel calculation using a simplified Monte Carlo method to treatment plan for scanned proton beams

**DOI:** 10.1120/jacmp.v17i2.5747

**Published:** 2016-03-08

**Authors:** Shohei Mizutani, Yoshihisa Takada, Ryosuke Kohno, Kenji Hotta, Ryohei Tansho, Tetsuo Akimoto

**Affiliations:** ^1^ Graduate School of Pure and Applied Sciences University of Tsukuba Tsukuba Ibaraki Japan; ^2^ Particle Therapy Division Research Center for Innovative Oncology National Cancer Center Hospital East Chiba Japan

**Keywords:** dose kernel calculation, simplified Monte Carlo method, treatment planning system, nasopharyngeal tumor case, proton pencil beam scanning

## Abstract

Full Monte Carlo (FMC) calculation of dose distribution has been recognized to have superior accuracy, compared with the pencil beam algorithm (PBA). However, since the FMC methods require long calculation time, it is difficult to apply them to routine treatment planning at present. In order to improve the situation, a simplified Monte Carlo (SMC) method has been introduced to the dose kernel calculation applicable to dose optimization procedure for the proton pencil beam scanning. We have evaluated accuracy of the SMC calculation by comparing a result of the dose kernel calculation using the SMC method with that using the FMC method in an inhomogeneous phantom. The dose distribution obtained by the SMC method was in good agreement with that obtained by the FMC method. To assess the usefulness of SMC calculation in clinical situations, we have compared results of the dose calculation using the SMC with those using the PBA method for three clinical cases of tumor treatment. The dose distributions calculated with the PBA dose kernels appear to be homogeneous in the planning target volumes (PTVs). In practice, the dose distributions calculated with the SMC dose kernels with the spot weights optimized with the PBA method show largely inhomogeneous dose distributions in the PTVs, while those with the spot weights optimized with the SMC method have moderately homogeneous distributions in the PTVs. Calculation using the SMC method is faster than that using the GEANT4 by three orders of magnitude. In addition, the graphic processing unit (GPU) boosts the calculation speed by 13 times for the treatment planning using the SMC method. Thence, the SMC method will be applicable to routine clinical treatment planning for reproduction of the complex dose distribution more accurately than the PBA method in a reasonably short time by use of the GPU‐based calculation engine.

PACS number(s): 87.55.Gh

## I. INTRODUCTION

Proton therapy has an advantage of dose localization that enables better dose conformation to a tumor and spares critical organs and normal tissues surrounding the tumor.^(1)^ Although a passive beam delivery technique has been used in proton therapy due to its stability of the irradiation

field and applicability to moving organs, the number of facilities adopting the pencil beam scanning (PBS) technique is increasing rapidly due to the inherent better dose conformity, the lack of need of manufacturing patient‐specific devices, and the lower ambient neutron dose.^2^, ^3^, ^4^ A number of facilities begin to apply the PBS technique to cancer treatment in moving organs like lung and liver by using a repainting technique and a gating technique.^5^, ^6^ Currently, a treatment planning system (TPS) for the PBS uses an analytical dose calculation method using a pencil beam algorithm (PBA) for proton therapy.^7^, ^8^ Although this algorithm allows short computation time and is suitable for dose calculation in homogeneous or moderately inhomogeneous media, the accuracy limitation appears in certain clinical sites with large density heterogeneity. The limitations arise from the algorithm of the PBA that the density heterogeneity is only considered on the single, straight central path of the pencil beam. In contrast, Full Monte Carlo (FMC) dose calculation takes into the basic physical processes in medium and tracks paths of individual primary protons and secondary particles. As a result, it has been considered as the most accurate calculation method in radiotherapy.^(9)^ However, since it takes much time for the FMC method to complete the dose calculation in clinical cases, it is difficult at present to apply the FMC calculation to routine clinical treatment planning. To reduce the calculation time, a number of faster Monte Carlo methods dedicated to proton therapy have been developed.^10^, ^11^, ^12^ One of such faster FMC codes for TPS in proton therapy, named VMCpro, was reported to be 35 times faster than the general purpose FMC code GEANT4 for simulations in a phantom with large inhomogeneities.^(10)^ The calculation time is approximately 30 to 75 times longer than that by the PBA with an original spot decomposed into 121 subspots.^8^, ^10^, ^11^ Therefore, further reduction of calculation time is desired.

A simplified Monte Carlo (SMC) method^12^, ^13^, ^14^, ^15^ has been developed to obtain dose calculation results more accurately than the PBA in target heterogeneities in a shorter calculation time. It tracks individual proton paths scattered in material and uses a measured depth‐dose distribution in water for dose calculation. The employed scattering model uses a multiple Coulomb scattering approximated as a Gaussian. The SMC method takes into account the effect of medium with the laterally different densities on dose distribution. Hotta et al.^(15)^ verified accuracy of the SMC method by comparing calculation results with dose distributions measured in an anthropomorphic phantom for the passive beam delivery system at the National Cancer Center Hospital East (NCCHE) in Japan. They found that the SMC method reproduced the measured dose distribution well, satisfying an accuracy tolerance of 3 mm and 3% in the gamma index analysis. The algorithm has been integrated in the clinical TPS of NCCHE for the passive beam delivery system. The NCCHE is now developing a PBS. Since the PBS delivery system requires an accurate and fast dose calculation engine, we have developed such a dose kernel calculation algorithm using the SMC method applicable to the dose optimization procedure for the PBS in the NCCHE.

We evaluated accuracy of the SMC calculation by comparing a dose distribution in an inhomogeneous phantom obtained by using the SMC method with dose distribution obtained by using the FMC method. In order to assess the usefulness of SMC calculation in clinical environment, we compared dose calculation results using the SMC method with those using the PBA method for three cases when the PBS would be used as a beam delivery method. Since it takes a reasonably short time for the SMC to complete dose calculation, it will have a possibility of being used for routine treatment planning.

## II. MATERIALS AND METHODS

**Figure 1:**
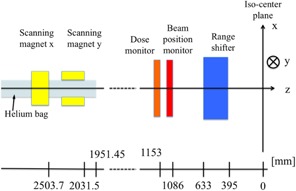
Schematic layout of the beam scanning delivery devices at NCCHE.

A multipurpose nozzle has been installed in the NCCHE. A PBS mode, as well as a beam‐wobbling mode, can be selected in this nozzle. Primary proton beam energy can be selected from one of 206 MeV, 192 MeV, and 176 MeV in the PBS mode at present. In the near future, more number of therapeutic beam energies will be available. In the present calculation study of clinical cases, we used 150 MeV as the primary proton energy using the passive beam delivery system, since 176 MeV proton beam has too long a range for clinical cases of head and neck. In the PBS mode, this nozzle uses a binary range shifter (RS) made from PMMA for the range shifting. The thickness of the range shifter can be controlled from 0 to 127 mm, with a resolution of 1 mm. The water‐equivalent thickness ratio of the PMMA is 1.16. Figure 1 shows the schematic layout of the beam scanning system at NCCHE. At present, the PBS mode is in commissioning phase. The beam‐wobbling mode is used for patient treatment concurrently.

### A. SMC calculation method

The SMC method calculates a dose distribution by tracking individual proton paths. It starts calculation at the entrance of patient body with initial beam parameters provided by the effective source model.^(7)^ The model parameters are calculated and prepared in advance for different combinations of the binary RS plates. Table 1 shows examples of the initial beam parameters for different binary RS thicknesses. The $\sigma_{\text{eff},x}$ and $\sigma_{\text{eff},y}$ in Table 1 are rms spatial beam spreads at the effective source point in X and Y directions, respectively. The $\sigma_{\text{eff},\theta x}$ and $\sigma_{\text{eff},\theta y}$ in Table 1 are rms angular spreads at the effective source point in X and Y directions, respectively. The spatial and angular distributions are assumed to be Gaussian. The $L_{\text{eff},x}$ and $L_{\text{eff},y}$ in Table 1 are distances between the effective beam source position and the isocenter in X and Y directions, respectively. In the SMC method, one million protons are generated for dose kernel calculation of each pencil beam. Each proton in a beam kernel is characterized by the position (x, y, z), direction in projection angles $\left(\theta_{x},\theta_{y}\right)$, residual range in water. It is assumed that the trajectory of each proton is determined only by the multiple Coulomb scattering with a scattering angles expressed as a normal random number with a standard deviation following the Highland's formula.^(16)^ For material in the body, we convert a CT value to water‐equivalent thickness of the voxel using a calibration table. Since we are using the water‐equivalent model, we calculate the rms scattering angle in the voxel as if the material is equivalent to water with the water‐equivalent thickness.^(13)^ The dose deposition in a voxel is obtained from a measured depth‐dose distribution in water using the water‐equivalent model.^(7)^ Shorter calculation time compared with FMC methods comes from simplification in which the dose deposition in materials is obtained by using the measured depth‐dose distribution in water, and ignoring nuclear reactions. Yet, use of the measured depth‐dose distribution for dose calculation implicitly includes energy losses due to nuclear collisions, energy deposition by secondary particles, loss of primary protons by nuclear interaction, and range straggling effect on average, as well as energy losses due to electron stopping. Such a simplification serves to reduce the calculation time of the dose deposition in a voxel. Accuracy of the SMC method was confirmed by experiments.^14^, ^15^ The estimated mean statistical error of the total dose calculation in the planning target volume (PTV) with the optimized spot weights was 0.57% rms of the maximum dose in the PTV.

**Table 1:** Examples of initial beam parameters

*RS thickness (mm)*	*2*	*32*	*64*	*96*
$\sigma_{\text{eff},x}(\text{mm})$	4.62	5.61	5.25	6.33
$\sigma_{\text{eff},y}(\text{mm})$	4.75	5.48	5.19	6.23
$\sigma_{\text{eff},\theta_{x}}(\text{mrad})$	5.95	17.08	25.82	38.37
$\sigma_{\text{eff},\theta_{y}}(\text{mrad})$	5.76	17.01	25.78	38.34
$L_{\text{eff},x}(\text{mm})$	989.40	491.00	599.70	501.38
$L_{\text{eff},y}(\text{mm})$	922.50	479.40	595.01	499.10

### B. Accuracy verification of the SMC method by comparison with the FMC method

**Figure 2:**
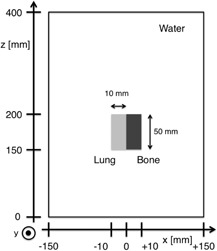
Numerical inhomogeneous phantom consisting of bone‐simulated material and lung‐simulated material immersed in water for the benchmark calculation.

To verify the accuracy of the SMC method, we compared a dose distribution for 206 MeV in water and that in a numerical inhomogeneous phantom obtained by the SMC method with those by the FMC method. As a FMC calculation, we used the PTSIM^(17)^ which is a simulation code for particle therapy built on the GEANT4 toolkit version 4.9.6, since it is considered accurate enough as a golden standard of dose calculation. As the numerical inhomogeneous phantom, we introduced an inhomogeneity in water of size $300\times 300\times 400\;{\text{mm}}^{3}$ (Fig. 2). The inhomogeneity consists of two cuboid blocks of size $10\times 20\times 50\;{\text{mm}}^{3}$ placed side by side. One of them consists from a bone‐simulated material $\left(\rho =1.575\;g\;{\text{cm}}^{-3}\right)$ and the other consists from a lung tissue‐simulated material $\left(\rho =0.217\;g\;{\text{cm}}^{-3}\right)$. The materials are defined in the PTSIM toolkit. The entrance faces of the two blocks are placed at a depth of 150 mm in water. The interface between the two blocks is placed on the beam central axis around which an initial pencil beam is generated. Such a selection of beam arrangement is considered to be the most challenging situation for pencil beam dose calculation algorithms. Dose distributions in the inhomogeneous phantom were calculated by the SMC and the FMC methods with 10^6^ generated protons using the same input data of the proton beam. The resulting laterally integrated depth‐dose curves and isodose curves have been compared. In this case, we used a laterally integrated depth‐dose curve in water calculated by the FMC method as the depth‐dose curve used in the SMC method. We take the voxel size of 1 mm in all directions.

### C. Simulation condition of clinical cases

For simulation, we have selected three clinical cases that have been treated at NCCHE using the proton beam with the passive beam delivery system. Two head and neck cases are labeled case A and B, and a lung and bronchus case is labeled case C in this article. All the cases use two‐beam port irradiation. To assess the usefulness of SMC calculations in the clinical environment, we compared the dose calculation results obtained by using the SMC method with those using the PBA method if the PBS would be used as a beam delivery method for the target. The software for dose calculation using the PBA method has been developed in‐house for the study based on the algorithm shown in Hong.^(7)^ All of these plans are SFUD (single‐field uniform dose) plans. Table 2 shows the plan details.

Spot geometry for each field is arranged so that spots are regularly spaced on 12 mm×12 mm grids in lateral directions considering the large beam size and by 5 mm water‐equivalent depth. For the CT input data, we take the in‐plane pixel sizes of either 1.17 or 1.88 mm depending on the case and slice separations of either 3 or 4 mm. The size of dose calculation grid is taken as the same as the CT voxel.

**Table 2:** Treatment plan information

	*Case A*	*Case B*	*Case C*
Tumor site	Nasopharynx	Maxillary Sinus	Lung
No. of fields	2 (0°, 10°)	2 (0°, 20°)	2 (0°, 330°)
No. of spots	757	1533	626
No. of PTV voxels^a^	28,600	88,549	8727

a ^a^ The voxel size of case A and B is $1.17\times 1.17\times 3\;{\text{mm}}^{3}$; the voxel size of case C is $1.88\times 1.88\times 4\;{\text{mm}}^{3}$.

## III. RESULTS & DISCUSSION

### A. Accuracy verification of the SMC method by comparison with the FMC method

**Figure 3:**
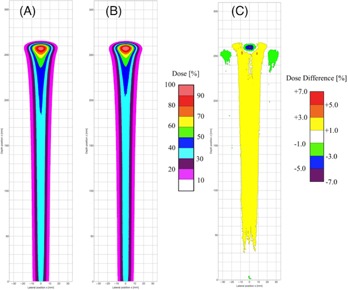
Isodose distributions in water obtained by the SMC method (a) and by the FMC method (b); (c) relative dose differences in water obtained by subtraction of the calculation result by the FMC method from that by the SMC method divided by the maximum dose in the FMC method.

Before proceeding to comparison of dose distributions in inhomogeneous phantom between the SMC and FMC methods, we compared dose distribution in water obtained by the SMC method with that by the FMC method to investigate the effect of different physics model employed in each method on the dose distribution. Figure 3 shows isodose distributions in water obtained by integrating the 3D dose distributions in the Y direction (see Fig. 2 for the coordinate system) perpendicular to the isodose plane (x‐z plane) and the difference distribution. The rms relative dose difference is 1.1% of the maximum dose in the region of 70 mm in width and 270 mm in depth. We found that the systematic dose difference can be attributed in part to ignorance of the lateral tail due to the nuclear reaction in the SMC method and in part to the different models^16^, ^18^ of the multiple Coulomb scattering employed by the SMC and FMC methods. The spatial deviation due to multiple Coulomb scattering in water calculated with the FMC method was found to be about 15% less than that with the SMC method. We notice the underdose region (the blue colored region) behind the Bragg peak in the SMC method, as shown in Fig. 3(c), since the SMC overestimates the lateral spread in this region. We also notice the lateral tail dose due to the nuclear elastic scattering shown as a green region near the Bragg peak in Fig. 3.

**Figure 4:**
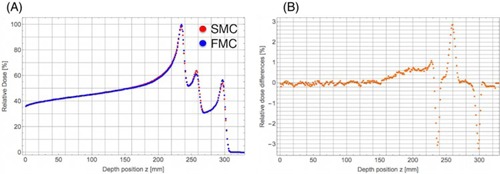
Laterally integrated depth dose in water‐equivalent distributions (a) in the numerical inhomogeneous phantom. Red and blue circles show calculation results obtained by the SMC and FMC methods, respectively. Relative dose in water‐equivalent differences distribution (b) subtracting dose calculated with the FMC method from that with SMC method divided by the maximum laterally integrated dose in water‐equivalent in the FMC method.

**Figure 5:**
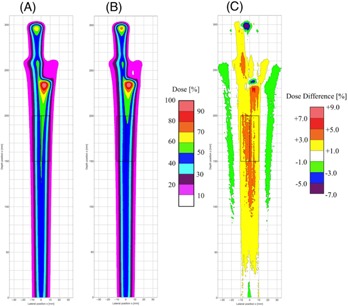
Isodose in water‐equivalent distributions (a) in the inhomogeneous phantom obtained by the SMC method and that obtained by the FMC method (b); (c) relative dose in water‐equivalent difference in the inhomogeneous phantom obtained by subtraction of the calculation result by the FMC method from that by the SMC method divided by the maximum dose in the FMC method.

In order to evaluate accuracy of the SMC method for practical cases, dose distributions obtained by the SMC and FMC methods have been calculated for the inhomogeneous numerical phantom consisting of a bone material and a lung material in water. Laterally integrated depth‐dose in water‐equivalent distributions in the phantom are shown in Fig. 4(a). The calculated doses in water equivalence are normalized by the dose in water at the Bragg peak position in Fig. 3 and we take the dose at the shallowest Bragg peak (z=234.5 mm) in the FMC method as 100%. We found good agreement of overall dose in water‐equivalent distributions between the SMC and FMC methods, although we noticed overestimation of calculated dose in a number of places with the SMC method. The shallowest Bragg peak is dominated by proton tracks penetrating through the bone, while protons penetrating through the lung slab produce the deepest Bragg peak (z=296.5 mm), and the protons escaping both slabs produce the middle peak (z=257.5 mm). We also notice small dose difference of about 0.6% in the region behind the bone slab and before the first peak. It may come from more reduction of primary protons in the bone slab due to the more nuclear reaction rate in the bone slab than in water in the FMC method, while the SMC method cannot reproduce the effect due to the adoption of the water‐equivalent model. And the rms relative dose in water‐equivalent difference in Fig. 4(b) is 0.8% of the maximum integrated dose for all points up to the depth of 310 mm. Relatively large dose differences have been found in the each distal falloff region of the Bragg peak due to the interpolation error of dose calculation in the SMC method. Excluding such distal falloff regions of the Bragg peak, the rms relative dose in water‐equivalent difference reduces to 0.5%. Figure 5 shows isodose in water‐equivalent distributions in the phantom obtained by integrating the 3D dose in water‐equivalent distributions in the direction perpendicular to the isodose plane calculated by both methods and the difference distribution. The rms relative dose in water‐equivalent difference is 1.3% of the maximum dose for all points in the region of 70 mm in width and 310 mm in depth. Note that different appearances of Fig. 5(c) and Fig. 3(c) come from dose normalization by the different maximum doses in the figures. Since the maximum dose in Fig. 5(c) is less than that in Fig. 3(c), more details of differences in lateral dose distribution can be seen in Fig. 5(c). Apart from the dose in water‐equivalent normalization by the different maximum doses, we notice that the difference in lateral dose in water‐equivalent distributions between the SMC and FMC methods in Fig. 5(c) is similar to that in Fig. 3(c). In summary, the overall dose in water‐equivalent distribution of the SMC method is in good agreement with that of the FMC.

### B. Dose kernel distribution

**Figure 6:**
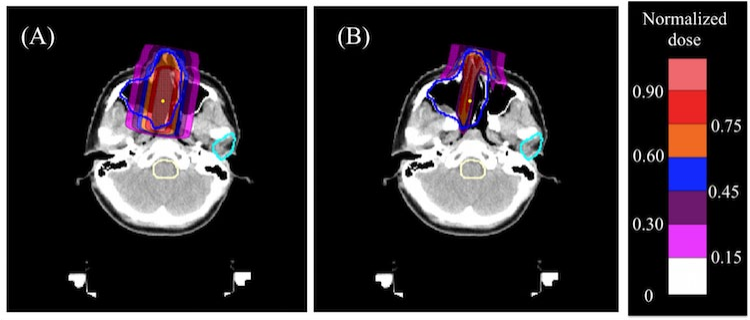
A single dose kernel distribution (a) calculated with the PBA method and (b) calculated with the SMC method. Blue and cyan lines are the PTV and the parotid gland, respectively; the yellow point is the spot position.

**Figure 7:**
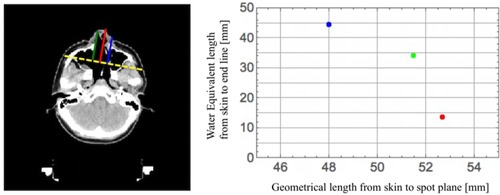
Water‐equivalent path lengths along different paths parallel to the beam central axis across the pencil beam. In the left figure, while the red line shows the beam central axis, blue and green lines show off‐axes slightly further away from central axis. The yellow dashed line shows a line (end line) passing through end points of the three paths. The line is perpendicular to three paths. The end point of the beam central axis is the spot position. The right graph indicates relation of the water‐equivalent path length to the geometrical length beam for the different three paths: the red circle corresponds to the beam central axis, the green circle corresponds to the off‐axis on the left, and the blue circle corresponds to the off‐axis on the right.

Figure 6(a) shows a dose kernel distribution obtained by the PBA calculation and Fig. 6(b) shows that obtained by the SMC calculation for the case A. For the dose calculations, we used the same initial beam parameters. Difference of the two dose kernel distributions can be noticed clearly. While the dose kernel calculated by the SMC method in the heterogeneous region reproduces the irregular dose distribution accurately, the PBA dose kernel fails to reproduce it. Since the PBA method determines the dose deposition and lateral spread based on materials along the beam central axis even for such a clinical situation involving large tissue heterogeneity, the dose kernel shows a symmetric dose distribution with respect to the central axis. Since lateral extent of beam kernels at NCCHE is very large (σ∼10 mm) due to the scattering in the binary RS, disregarding density heterogeneities across a pencil beam can lead to considerable errors in the dose estimation. Figure 7 indicates water‐equivalent path lengths parallel to the beam central axis across the pencil beam. While the beam central axis (red line) has the longest geometric length from skin to spot position, it has the shortest water‐equivalent length compared with the other two adjacent off‐axes. Thus, real proton paths are intensely affected by the off‐axis materials. For instance, a proton entering into the off‐axis materials region stops at a shallower position than the proton entering along the central axis. Reflecting the largely varying water‐equivalent thicknesses of off‐axes around the central beam axis, the dose distribution calculated by the SMC method is irregular. The influence of air cavities and bone structure on the dose distribution can be clearly observed.

### C. Optimized dose distribution

**Figure 8:**
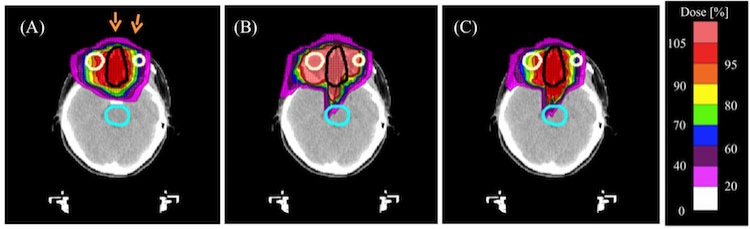
Calculated dose distributions obtained by (a) PBA TP, (b) SMC recalculation, and (c) SMC TP in an isocenter plane in the case A combining two irradiation fields from two beam directions. The black line shows an outline of the PTV. The light blue line is the outline of the brainstem. The yellow lines are the outlines of right and left eyeballs. The orange arrows are the field direction.

**Figure 9:**
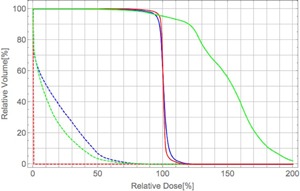
DVHs for the PTV and the brainstem in the case A for two fields plan. Red, green, and blue lines are DVHs for the PBA TP, SMC recalculation, and SMC TP, respectively. Solid and dashed lines show DVHs for the PTV and DVHs for brainstem regions, respectively.

Figures 8(a) and (b) show the dose distribution calculated with the PBA dose kernels with the spot weights obtained by optimization with the PBA method (named ‘PBA TP’), and that calculated with the SMC dose kernels with the same spot weights (named ‘SMC recalculation’) in the case A, respectively. Figure 8(c) shows the dose distribution calculated with the SMC dose kernels with the spot weights obtained by optimization with the dose kernels calculated by using the SMC method (named ‘SMC TP’). The dose‐volume histograms (DVHs) for the PTV and the brainstem corresponding to the dose distributions in Fig. 8 are shown in Fig. 9. Solid and dashed lines show DVHs for the PTV and DVHs for the brainstem, respectively. Red, green, and blue lines denote DVHs for the PBA TP, SMC recalculation, and SMC TP, respectively. Dose calculation result of the PBA TP shows an apparently homogeneous distribution in the PTV. In contrast, dose calculation result of the SMC recalculation shows that large excess doses are given in the large portion of the PTV, as shown as the green solid line of DVH for the PTV in Fig. 9. We also notice that, while no dose is apparently deposited in the brainstem for the PBA TP, the dose is really deposited in the brainstem for the SMC recalculation and the SMC TP, as shown in Fig. 8 and as shown as dashed lines in Fig. 9. It may have a risk of increasing complication of the organ at risk (OAR).

**Figure 10:**
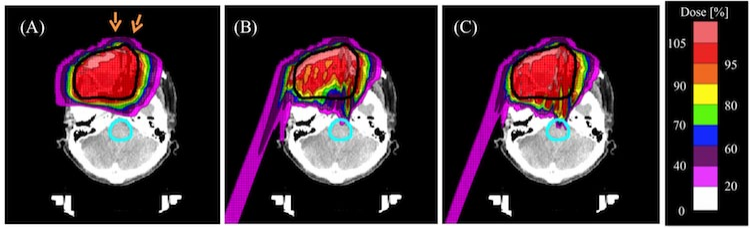
Calculated dose distributions obtained by (a) PBA TP, (b) SMC recalculation, and (c) SMC TP in an isocenter plane in the case B combining two irradiation fields from two beam directions. The black line shows an outline of the PTV. The light blue line is the outline of the brainstem. The orange arrows are the field direction.

**Figure 11:**
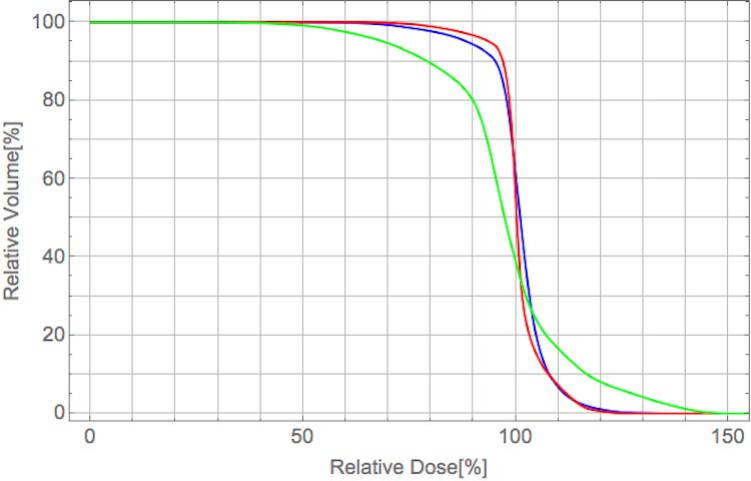
DVHs for the PTV in the case B for two fields plan. Red, green, and blue lines are DVHs for the PBA TP, SMC recalculation, and SMC TP, respectively.

Figures 10and 11 show the dose distributions and DVHs for the PTV of the PBA TP, SMC recalculation, and SMC TP in the case B, respectively. While the dose calculation result of the PBA TP apparently satisfies a criterion of more than 90% of the prescription dose in PTV $\left(D_{95}=93.8\%\right)$, as shown as the red line in the Fig. 11, that of the SMC recalculation shows that a largely inhomogeneous dose distribution is given in the PTV, and the $D_{95}$ of the PTV reduces to 68.9%, as shown in Fig. 10(b) and the green line in Fig. 11. In spite of such a difficult clinical case, the $D_{95}$ increases to 88.5% of prescription dose in the PTV by using SMC TP, as shown in Fig. 11. Since the lateral extent of the pencil beam kernel at NCCHE is very large, there is a trade‐off between dose coverage of the target volume and dose reduction in the critical organ. If we would require the strict clinical criterion that 95% of the PTV should be irradiated with 100% of the prescription dose, a treatment plan with an additional beam port from the direction of 330° would be necessary at the expense of more dose to the normal tissue.

**Figure 12:**
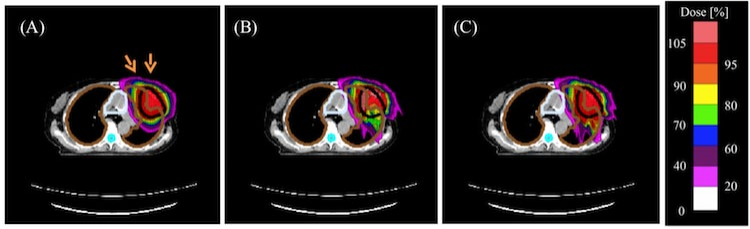
Calculated dose distributions obtained by (a) PBA TP, (b) SMC recalculation, and (c) SMC TP in an isocenter plane in the case C combining two irradiation fields from two beam directions. The black line shows an outline of the PTV. The brown lines are outlines of right and left lungs. The orange arrows are the field direction.

**Figure 13:**
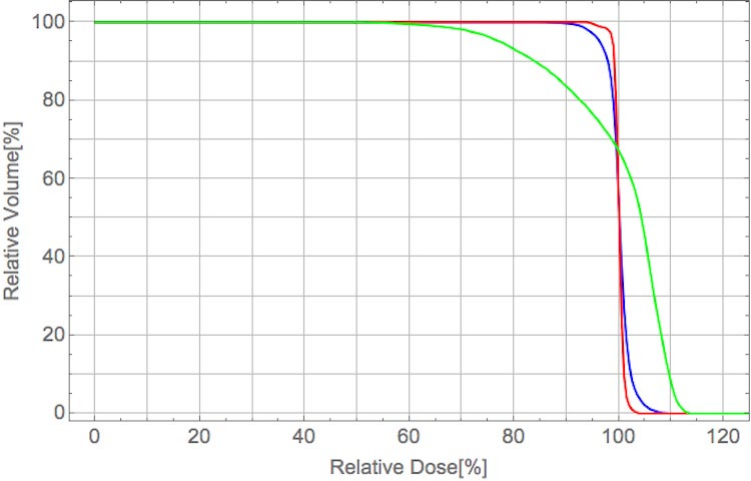
DVHs for the PTV in the case C for two fields plan. Red, green, and blue lines are DVHs for the PBA TP, SMC recalculation, and SMC TP, respectively.

Figures 12 and 13 show the dose distributions and DVHs for the PTV of the PBA TP, SMC recalculation, and SMC TP in the case C (tumor in the lung and bronchus), respectively. The dose distribution obtained by the SMC recalculation has an underdose portion in the overlapping region of the PTV and the lung, as shown in Fig. 12(b) and the green line in Fig. 13. In contrast, the dose distribution obtained by the SMC TP has a homogeneous dose distribution in the PTV, as shown in Fig. 12(c) and the blue line in Fig. 13.

By showing comparisons of dose distributions between the PBA method and the SMC method for three clinical cases, we find that the treatment plan obtained by the PBA method will have a risk of radiation hazard and/or local recurrence for the target and a risk of possible complications for OARs for complex clinical cases.

### D. Calculation time

We implemented the SMC method in the dose calculation by parallel computation on a 3.7 GHz Quad‐Core Intel Xeon Processor E5 (Intel Corporation, Santa Clara, CA). We used the Intel C++ compiler (Intel Parallel Studio XE 2015 Composer Edition for C++ OS X). We parallelized the part of dose calculation by OpenMP. The calculation time required for obtaining dose kernels by the SMC method was approximately 2116 s on the central processing unit (CPU) for 10^6^ primary protons per a dose kernel in the case A (total number of calculated dose kernels is 757). Calculation time required for obtaining both the dose optimization and full dose calculation with the optimized spot weights is approximately 232 s on the CPU in this case. Thus, calculation time required for obtaining dose kernels accounts for about 90% of the total calculation time. The time required for calculating all dose kernels by the SMC method is approximately 16.2 times longer than that by the PBA with an original spot decomposed into 121 subspots. Thus the SMC method is estimated to be faster than the VMCpro by a factor of 1.9 to 4.6. The dose calculation time depends on the size of PTV. If the dose calculation using the SMC method applies to the larger PTV volume of about 1000 ml (total number of calculated dose kernels is 4400), the calculation time is estimated to be approximately 12,273 s. On the other hand, Kohno et al.^(19)^ have implemented the SMC method on graphics processing unit (GPU) architecture under the computer‐unified device architecture platform (NVIDIA) developed by Nvidia Corp. (Santa Clara, CA) for a passive beam delivery system. Accordingly, we have calculated a dose distribution using the SMC method in the case A on the GPU when the PBS would be used as a beam delivery method. As a hardware platform, the GPU was located on a single graphics card (Tesla K40, Nvidia) with 12 GB of global memory. The GPU card holds

2880 CUDA cores. The calculation time required for obtaining the dose kernels was 133 s on the GPU‐based calculation engine when 10^6^ primary protons were generated per a dose kernel. Thus, the GPU‐based SMC calculation speed for dose kernels calculation is about 16 times faster than the CPU‐based one. Calculation time required for both dose optimization and full dose calculation with the optimized spot weights was 44 s on the GPU in this case. Thus, calculation time required for obtaining the dose kernels accounts for about 75% of the total calculation time on the GPU. Therefore, we expect the total calculation time of approximately 12.8 min on the GPU‐based calculation engine for the target volume of 1000 ml. The time can be considered reasonably short for routine clinical uses.

## IV. CONCLUSIONS

We have developed a dose kernel calculation algorithm using the SMC method applicable to the spot scanning system recently installed in the NCCHE in Japan. Calculation using the SMC method is faster than that using the general‐purpose FMC code by three orders of magnitude. In addition, the SMC method was found to be faster than the existing fast Monte Carlo method. Another advantage of the SMC method is easy implementation since it can use the same input data for the PBA calculation. We evaluated accuracy of the SMC method by comparison with the FMC method. We clarified the effect of model difference of the SMC and FMC methods on the dose calculation. Overall, the dose distribution obtained by the SMC method was in good agreement with that obtained by the FMC method in the inhomogeneous phantom. To assess the usefulness of SMC calculations in the clinical environment, we compared a treatment planning result using the SMC method with that using the PBA method for three cases when the PBS would be used as a beam delivery method. The results show that the SMC method can reproduce the complex dose distribution more accurately in a reasonably short time than the PBA method. Since the treatment plan using dose kernels obtained by the PBA method provides inaccurate dose distribution for clinical cases with heavily heterogeneous structure, it will increase clinical risks. Therefore, we should replace it by a treatment plan using the SMC dose kernels as soon as possible.

## ACKNOWLEDGMENTS

We appreciate SHI Accelerator Service Ltd. and Health Labor Sciences Research Grant (No. 26270701) for their support with this study.

## COPYRIGHT

This work is licensed under a Creative Commons Attribution 4.0 International License.
